# What is the best combination treatment with transarterial chemoembolization of unresectable hepatocellular carcinoma? a systematic review and network meta-analysis

**DOI:** 10.18632/oncotarget.20119

**Published:** 2017-08-10

**Authors:** Hui Xie, Haipeng Yu, Shengtao Tian, Xueling Yang, Ximing Wang, Zhao Yang, Huaming Wang, Zhi Guo

**Affiliations:** ^1^ Department of Interventional Therapy, Tianjin Medical University Cancer Institute and Hospital, National Clinical Research Center for Cancer, Key Laboratory of Cancer Prevention and Therapy, Tianjin's Clinical Research Center for Cancer, Tianjin 300070, China; ^2^ Department of Interventional Therapy, 302 Hospital of People's Liberation Army, Beijing 100039, China

**Keywords:** transarterial chemoembolization, TACE, unresectable hepatocellular carcinoma, combination treatment, network meta-analysis

## Abstract

**Objective:**

To assess the comparative efficacy and safety of combination treatment with transarterial chemoembolization (TACE) for patients with unresectable hepatocellular carcinoma (HCC) through a systematic review and network meta-analysis and to identify what is the best combination treatment with TACE.

**Materials and Methods:**

A network meta-analysis was used to identify evidence from relevant randomized controlled trials. We searched databases for publications up to June 2017. The prespecified primary efficacy outcomes were treatment response and 6-month to 3-year overall survival (OS), while the secondary efficacy outcomes were 1- and 2-year disease-free survival (DFS); safety outcomes were advance effects of combination treatment. We conducted pairwise meta-analyses using a random-effects model and then performed random-effects network meta-analyses.

**Results:**

A total of 48 trials were eligible (50 analyses), involving 5627 patients and 19 treatment arms. In comparison with other types of combination therapy arms, network meta-analysis disclosed that TACE + three-dimensional conformal radiotherapy, TACE + percutaneous ethanol injection, TACE + percutaneous microwave coagulation therapy, TACE + percutaneous acetic acid injection, and TACE + sorafenib were the more effective methods in treatment response, 6-month to 3-year OS, and 1–2 year DFS; the adverse effects of TACE + sorafenib were serious. The study was registered with PROSPERO, number CRD42017071102.

**Conclusions:**

When considering the efficacy, combination therapy with TACE seemed to offer clear advantages for patients with unresectable HCC. TACE + Three-dimensional conformal radiotherapy, TACE + Percutaneous ethanol injection, TACE + Percutaneous microwave coagulation therapy, and TACE + Percutaneous acetic acid injection are likely the best options to consider in the application of combination treatment.

## INTRODUCTION

Hepatocellular carcinoma (HCC) is one of the most common cancers and has the dismal outcome of cancer-related death [1]. The incidence of HCC has increased dramatically in the past decade, making HCC the fifth most common cancer in the world currently. Incidence rates continue to increase rapidly for liver cancer, by about 3% per year in women and 4% per year in men, although rates have begun to decline in adults younger than 50 years of age [2]. It is recognized that only a small proportion of patients with early-stage HCC may benefit from radical options, such as surgical resection and orthotopic liver transplantation. Although hepatic resection offers a first-line hope for patients suffering from HCC, only a small proportion (10–15%) of HCC patients are suitable [3]. However, surgical resection is not the first treatment choice for HCC patients with large lesions or poor liver function.

Palliative care and management including transarterial chemoembolization (TACE) is prescribed for most HCC patients to prevent and relieve suffering and improve their quality of life. TACE is a standard minimally invasive procedure developed for HCC patients who are not eligible for complete resection [4]. TACE involves the injection of a chemotherapeutic agent, which induces selective vascular embolization and blocks the arteries, hence triggering tumor infarction and necrosis [5–6] and the combination of fluorouracil, cisplatin , mitomycin or epirubicin in the TACE treatment was the most common regimen [7].Patients with large and multiple lesions exceeding the Milan criteria have been widely treated by TACE, which has been proven to improve the survival of those patients [8–10]. However, TACE may further affect liver functions and damage the hepatic arterial system. As a result, TACE is not appropriate for patients with poor liver functions, particularly those with cirrhosis, which are the limitations of TACE [11].

However, it is usually difficult to achieve complete necrosis of the target lesion by TACE alone because of the intracapsular or extracapsular invasion of unresectable HCC and viable tumor cells remaining after treatment [12]. Thus, repeated procedures are needed to achieve better results, although not much survival benefit has been gained with TACE alone [13–14]. Moreover, three-dimensional conformal radiotherapy (3DCRT) [15], radiofrequency ablation (RFA) [16–17], percutaneous ethanol injection (PEI) [18], percutaneous acetic acid injection (PAI) [19], percutaneous microwave coagulation therapy (PMCT) [20], drug-eluting bead transarterial chemoembolization (DEB-TACE) [21], and sorafenib [22] have also been shown to be highly effective in the treatment of HCC. Studies have also suggested that TACE combined with other percutaneous techniques and targeted therapy drugs may improve survival [23–24].

Although this combination therapy has been used in patients suffering from unresectable HCC, the current data on therapeutic effects are controversial, and its clinical role has not been decided. To further explore these issues and to identify the best combination treatment with TACE, we performed a network meta-analysis of all available clinical trials of patients with unresectable HCC. No previous reviews [18, 25–27] have provided a comprehensive overview with meta-regressions and network meta-analyses.

## RESULTS

### Description of the network and patients

**Figure 1:**
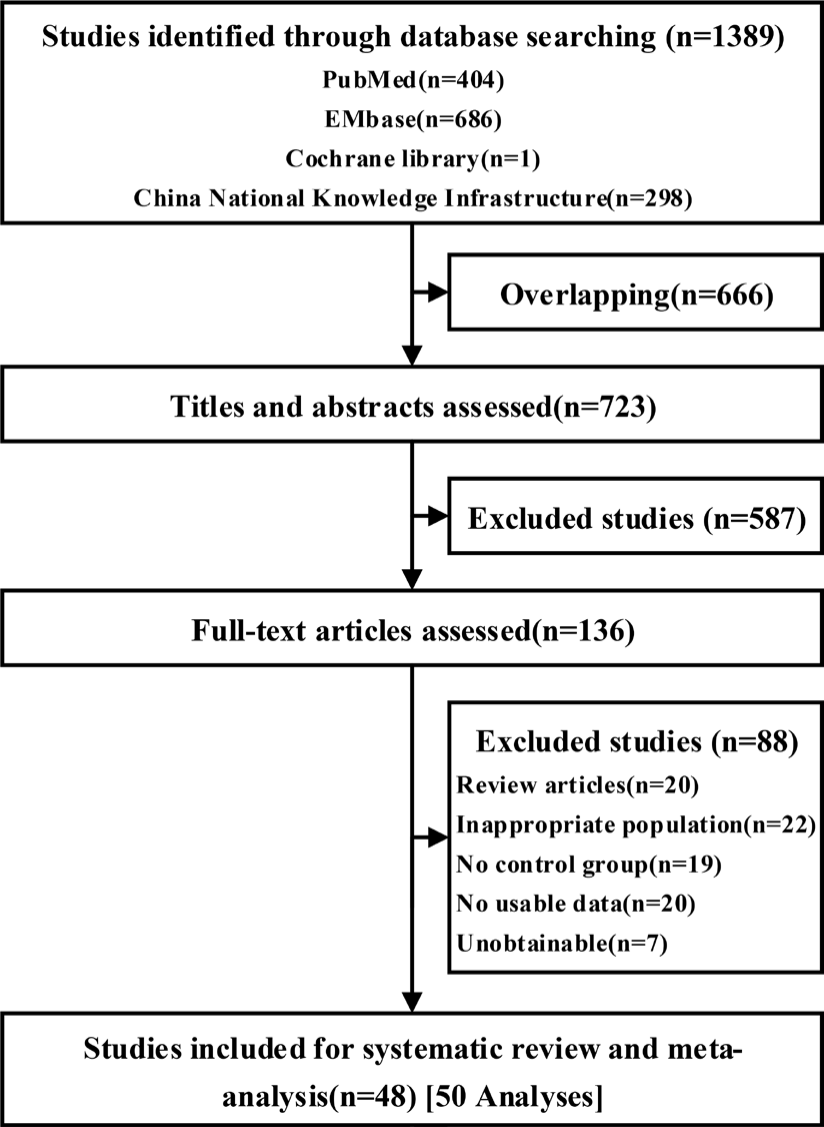
Flow of studies through the review process for the systematic review and network meta-analysis

In total, 1389 citations were retrieved from the databases; after removing duplicates, 723 citations were screened on title and abstract; 587 were excluded from further analysis. A total of 123 citations were included for full-text analysis. The network consists of 48 trials and 5627 patients, which were included in the standard meta-analysis. Because of a factorial design in two trials, these 48 trials were split into 50 analyses. There were 19 different treatments: (1) TACE, (2) DEB-TACE; (3) TACE + sorafenib; (4) TACE + stereotactic body radiation therapy (TACE + SBRT); (5) TACE + TACE + PMCT; (6) TAI; (7) TACE + Licartin; (8) selective internal radiotherapy (SIRT); (9) TACE + RFA); (10) RFA; (11) TACE + brivanib; (12) TACE + 3DCRT; (13) TACE + amiodarone; (14) TACE+ interferon-ɑ (TACE + IFN); (15) TACE + PEI; (16) TACE + radiotherapy therapy (TACE + RT); (17) TACE + PAI; (18) percutaneous acetic acid injection (PAI); (19) high-dose hepatic arterial infusion chemotherapy (HAIC). Figure 1 shows the screening flowchart.

Table 1 summarizes the differences in the fundamental characteristics between the two treatment arms (see full characteristics information in Supplementary Table 1). The statistics showed that the two groups had similar baseline results in age, gender, tumor stage, tumor size, Child-Pugh, ECOG status, HBV infection rate, HCV infection rate, and number of tumors. The assessments of study quality are presented in Supplementary Table 2, and the NOS scale score result shows that all included studies had an acceptable quality.

**Table 1:** Characteristics of baseline in patients associated with Treatment arm I vs Treatment arm II

	Treatment arm I vs II (OR, 95%CI)	Heterogeneity
**Age (year)**	0.015 (−0.08, 0.11)^*^	*P* = 0.890, *I*^*2*^= 0.0%
**Male**	0.98 (0.84, 1.15)	*P* = 0.417, *I*^*2*^ = 3.0%
**Tumor stage (I–II/III–IV)**	0.90 (0.61, 1.33)	*P* = 0.210, *I*^*2*^ = 23.8%
**Tumor size (cm) (< 5/5-/ < 10/10-)**	1.01 (0.83, 1.23)	*P* = 0.903, *I*^*2*^ = 0.0%
**Child-Pugh (A/B–C)**	0.99 (0.78, 1.26)	*P* = 0.063, *I*^*2*^ = 31.7%
**ECOG status (0/1)**	1.30 (0.95, 1.78)	*P* = 0.068, *I*^*2*^ = 45.1%
**HBV (+/−)**	0.97 (0.81, 1.15)	*P* = 0.971, *I*^*2*^ = 0.0%
**HCV (+/−)**	0.95 (0.78, 1.18)	*P* = 0.999, *I*^*2*^ = 0.0%
**Number of tumors (single/multiple)**	0.72 (0.49, 1.06)	*P* = 0.000, *I*^*2*^ = 70.2%

^*^Standardized mean difference;

**Figure 2:**
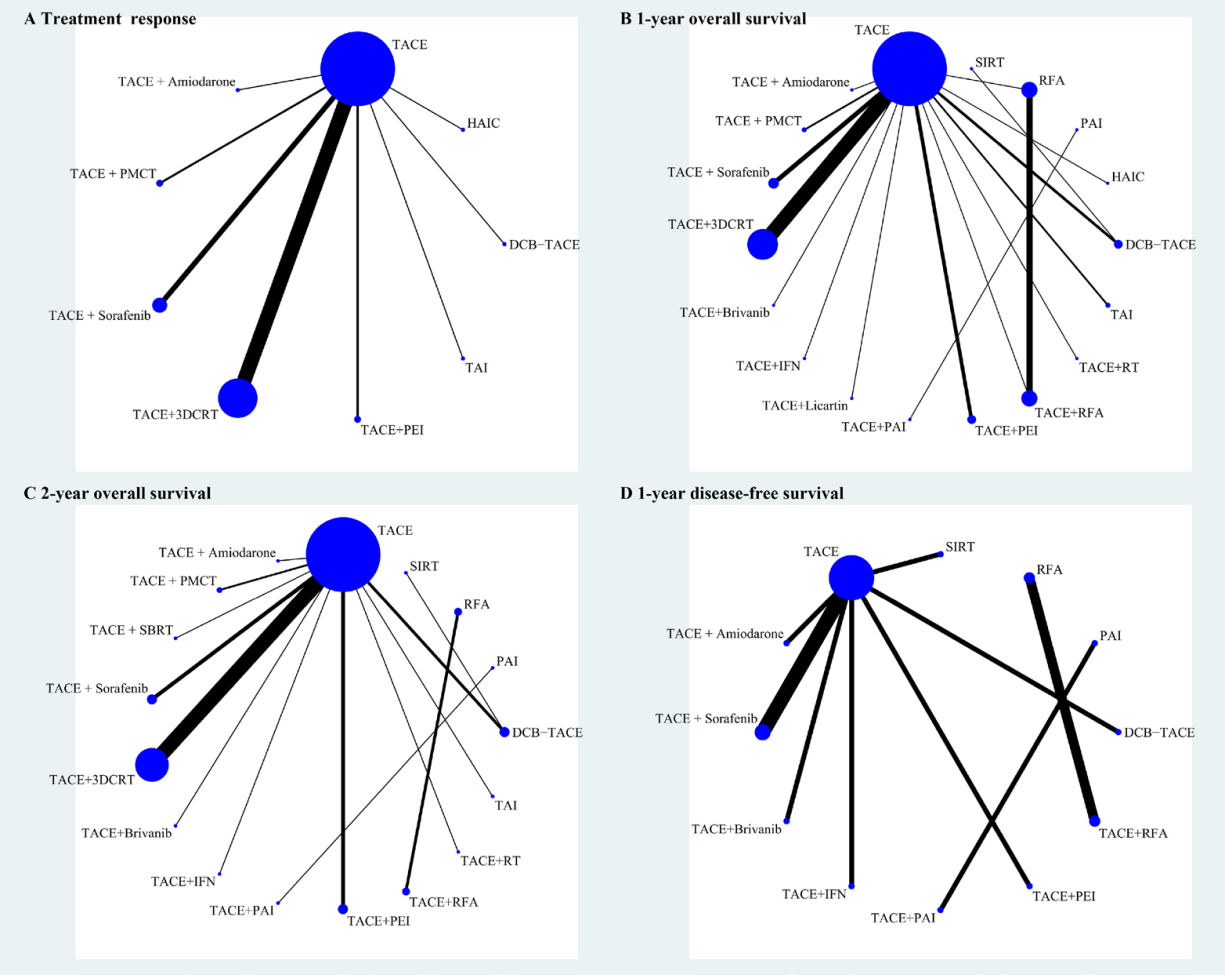
Network of eligible comparisons for treatment response (**A**), 1-year overall survival (**B**), 2-year overall survival (**C**) and 1-year disease-free survival (**D**). The width of the lines is proportional to the number of trials comparing every pair of treatments, and the size of every circle is proportional to the number of randomly assigned participants (sample size). Abbreviations: 3DCRT, three-dimension eonformal radiotherapy; DEB-TACE, drug-eluting bead transarterial chemoembolization; HAIC, high-dose hepatic arterial infusion chemotherapy; IFN, interferon-ɑ; PAI, percutaneous acetic acid injection; PEI, percutaneous ethanol injection; PMCT, percutaneous microwave coagulation therapy; RFA, radiofrequency ablation; RT, radiotherapy therapy; SBRT, stereotactic body radiation therapy; SIRT, selective internal radiotherapy; TACE, transarterial chemoembolization; TAI, transarterial infusion chemotherapy.

Figure 2 displays the network weight of eligible comparisons for treatment response (A), 1-year OS (B), 2-year OS (C), and 1-year DFS (D) displaying the available direct comparisons and network of the trials. Table 2 summarizes the numbers of patients with unresectable HCC according to study treatment. Patients were grouped by different treatment arms (most trials only had two arms). More than half of our included trials compared the efficacy and safety of TACE plus other treatment with TACE alone.

**Table 2:** Number of patients with unresectable hepatocellular carcinoma according to study treatment

Treatment arm I	Treatment arm II	Number of analyses	Patients(I/II)
TACE + 3DCRT	TACE	16 [38, 42, 44–45, 49–50, 55, 57–58, 62–64, 66–69]	615/645
TACE + Sorafenib	TACE	6 [29, 39, 41, 48, 51, 53]	528/660
DCB-TACE	TACE	3 [28, 31, 52]	154/239
TACE + PMCT	TACE	2 [32, 46]	102/116
TACE+Licartin	TACE	1 [34]	167/174
TACE+Brivanib	TACE	1 [37]	249/253
TACE + Amiodarone	TACE	1 [47]	13/14
TACE+IFN	TACE	1[59]	108/108
TACE+RFA	TACE	1 [65]	24/11
TACE+PEI	TACE	4 [70, 73–75]	102/100
TACE+RT	TACE	1 [71]	54/60
TACE + SBRT	TACE	1 [30]	44/52
TACE + PAI	PAI	1 [72]	53/55
TACE	TAI	2 [33, 60]	178/177
DCB-TACE	SIRT	1 [35]	12/12
TACE+RFA	RFA	7 [36, 40, 43, 56, 61, 65]	270/235
HAIC	TACE	1 [54]	31/36

3DCRT, three—dimension eonformal radiotherapy; DEB-TACE, drug-eluting bead transarterial chemoembolization; HAIC, high-dose hepatic arterial infusion chemotherapy; IFN, interferon-ɑ; PAI, percutaneous acetic acid injection; PEI, percutaneous ethanol injection; PMCT, percutaneous microwave coagulation therapy; RFA, radiofrequency ablation; RT, radiotherapy therapy; SBRT, stereotactic body radiation therapy; SIRT, selective internal radiotherapy; TACE, transarterial chemoembolization; TAI, transarterial infusion chemotherapy.

### Primary efficacy outcome - Treatment response

The network meta-analysis suggested that, in comparison with TACE treatment alone, DCB-TACE ranked the lowest for efficacy (OR: 0.41, 95% CI: 0.09–1.90); TACE + PEI ranked the best for the efficacy of treatment response (6.72, 1.43–31.60), followed by TACE + sorafenib (5.42, 2.60–11.30), TACE + 3DCRT (3.10, 2.04–4.72), TACE + PMCT (3.00, −1.11–8.12), HAIC (15.13, 0.00–6.72e + 6), TAI (15.13, 0.00–6.72e + 6), and TACE + amiodarone (1.67, 0.26–10.91).

**Figure 3:**
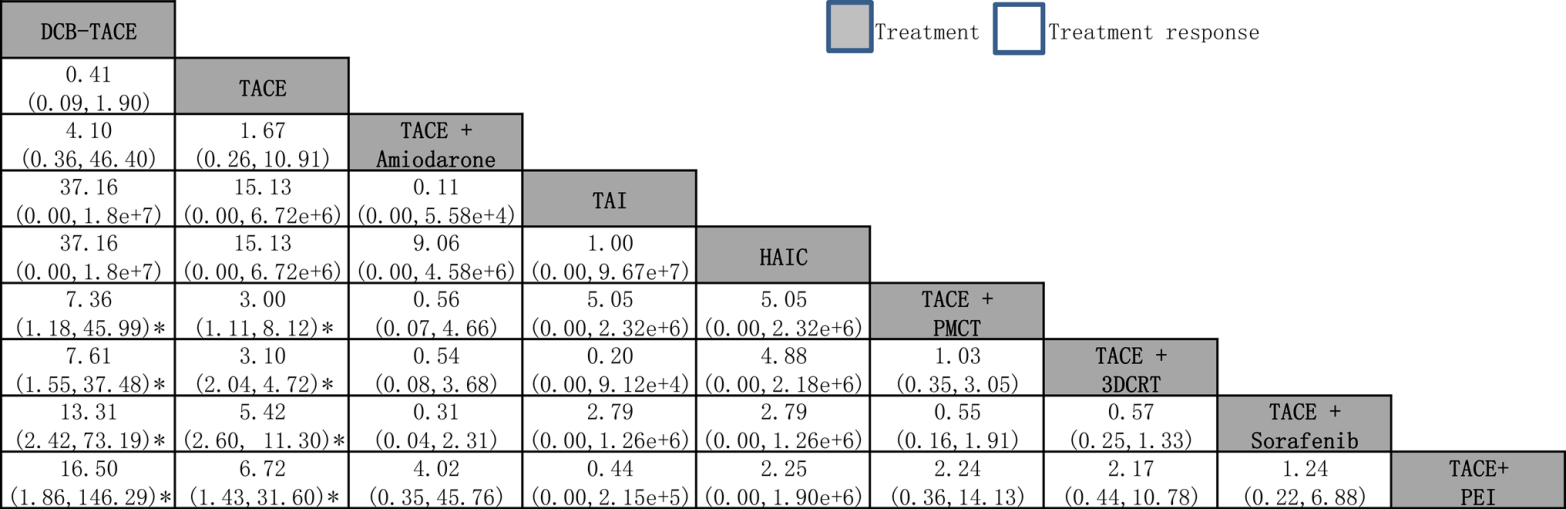
Network meta-analysis of treatment response Treatments are reported in order of survival rate ranking according to SUCRA of treatment response. Comparisons should be read from left to right. The estimate is located at the intersection of the column-defining treatment and the row-defining treatment. For treatment response, an OR value above 1 favors the treatment arm. ^*^Result with significant difference. Abbreviations: DEB-TACE, drug-eluting bead transarterial chemoembolization; HAIC, high-dose hepatic arterial infusion chemotherapy; PEI, percutaneous ethanol injection; PMCT, percutaneous microwave coagulation therapy; TACE, transarterial chemoembolization; TAI, transarterial infusion chemotherapy.

When we assessed the comparative efficacy, TACE + PEI was superior to all other treatment arms. However, the other treatment arms did not reach significance. Treatment arms were comparable with each other for improved treatment responses, with no significant differences found (Figure 3). The comparison-adjusted funnel plot of treatment response was not suggestive of any publication bias (Supplementary Figure 1).

### Primary efficacy outcome - OS

#### 6-month OS

The network meta-analysis suggested that, compared with TACE treatment alone, TACE+3DCRT was ranked best for improving 6-month OS (4.95, 1.53–15.96), followed by TACE + IFN (2.49, 1.04–5.99), TACE + Licartin (1.89, 1.20–2.97), TACE + PMCT (3.22, 0.66 –15.76), HAIC (2.33, 0.86–6.29), TACE + RFA (2.03, 0.47–8.74), TACE + sorafenib (1.76, 0.00–8.08e+5), SIRT (1.76, 0.00–8.08e+5), TACE + amiodarone (1.75, 0.00–7.75e+5), DEB-TACE (1.75, 0.00–7.75e+5), TACE + PEI (1.64, 0.48–5.61), TACE + brivanib (1.04, 0.70–1.55), RFA (0.89, 0.20–2.98), and TAI (0.01, 0.00–0.04).

**Figure 4:**
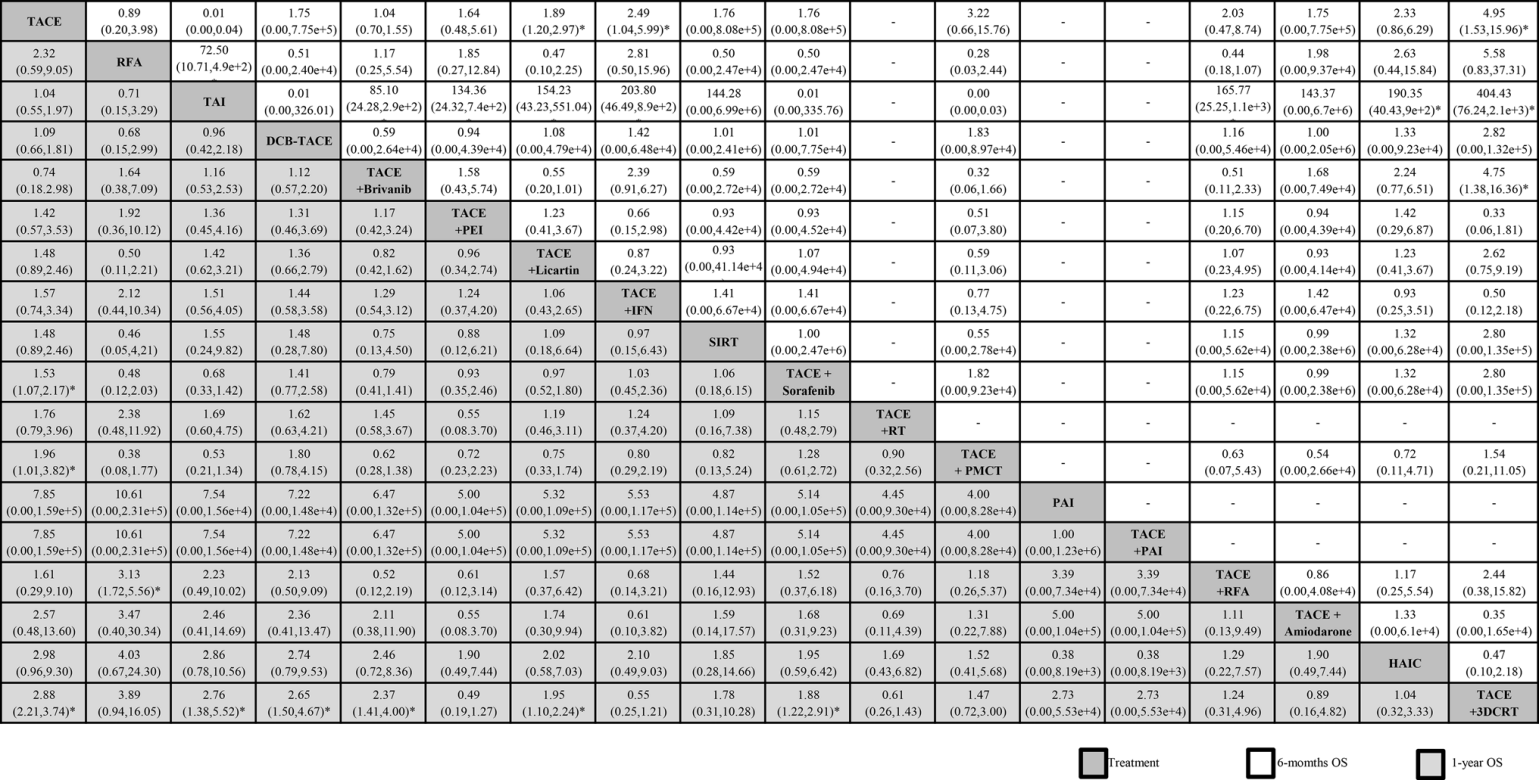
Network meta-analysis of 6-months OS and 1-year OS Treatments are reported in order of survival rate ranking according to SUCRA of treatment response. Comparisons should be read from left to right. The estimate is located at the intersection of the column-defining treatment and the row-defining treatment. For OS, an OR value above 1 favors the treatment arm. ^*^Result with significant difference. Abbreviations: 3DCRT, three—dimension eonformal radiotherapy; DEB-TACE, drug-eluting bead transarterial chemoembolization; HAIC, high-dose hepatic arterial infusion chemotherapy; IFN, interferon-ɑ; PAI, percutaneous acetic acid injection; PEI,percutaneous ethanol injection; PMCT, percutaneous microwave coagulation therapy; RFA, radiofrequency ablation; RT, radiotherapy therapy; SIRT, selective internal radiotherapy; TACE, transarterial chemoembolization; TAI, transarterial infusion chemotherapy.

When we assessed the comparative efficacy, TACE + 3DCRT was superior to all other treatment arms. However, except for the TACE + 3DCRT arm, TACE + IFN arm, and TACE + Licartin arm, the other treatment arms did not reach significance. Treatment arms were comparable with each other for improving 6-month OS, with a significant difference found in the TACE + 3DCRT vs TAI group (404.43, 76.24–2.1e+3), TACE + 3DCRT vs TACE + brivanib group (4.75, 1.38–16.36), and HAIC vs TAI group (190.35, 40.43–9e+2) (Figure 4).

#### 1-year OS

The network meta-analysis suggested that, compared with TACE treatment alone, TACE + 3DCRT was ranked best for improving 1-year OS (2.88, 2.21–3.74), followed by HAIC (2.98, 0.96–9.30), TACE + amiodarone (2.57, 0.48–13.60), TACE + RFA (1.61, 0.29–9.10), TACE + PAI (7.85, 0.00–1.59e+5), PAI (7.85, 0.00–1.59e+5),TACE + PMCT (1.96, 1.01–3.82),TACE + RT (1.76, 0.79–3.96), TACE + sorafenib (1.53, 1.07–2.17), SIRT (1.48, 0.89–2.46), TACE + IFN (1.57, 0.– 3.34), TACE + Licartin (1.48, 0.89–2.46), TACE + PEI (1.42, 0.57–3.53), TACE + brivanib (0.74, 0.18–2.98), DEB-TACE (1.09, 0.66–1.81), TAI (1.04, 0.55–1.97), and RFA (2.32,0.59–9.05).

When we assessed the comparative efficacy, TACE + 3DCRT was superior to all other treatment arms. However, except for the TACE + 3DCRT arm, TACE+PMCT arm, and TACE + sorafenib arm, the other treatment arms did not reach significance. Treatment arms were comparable with each other for improving 1-year OS, with a significant difference found in the TACE + 3DCRT vs TACE + sorafenib group (1.88, 1.22–2.91), TACE + 3DCRT vs TACE + Licartin group (1.95, 1.10–2.24), TACE + 3DCRT vs TACE + brivanib group (2.37, 1.41–4.00), TACE + 3DCRT vs DEB-TACE group (2.65, 1.50–4.67), and TACE + 3DCRT vs TAI group (2.76, 1.38–5.52) (Figure 4).

**Figure 5:**
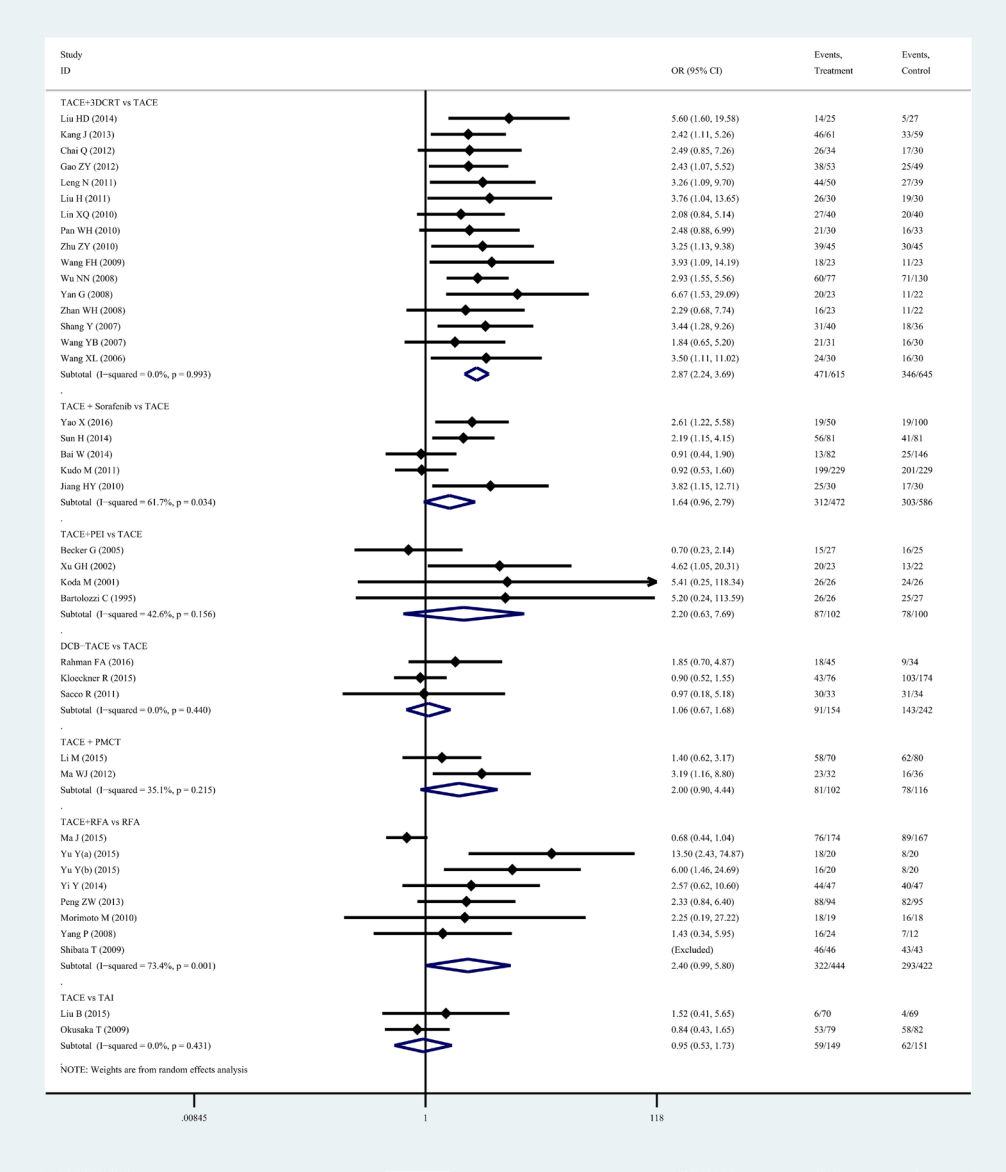
Overall 1-year survival for patients with unresectable hepatocellular carcinoma Abbreviations: 3DCRT, three—dimension eonformal radiotherapy; DEB-TACE, drug-eluting bead transarterial chemoembolization; PEI, percutaneous ethanol injection; PMCT, percutaneous microwave coagulation therapy; RFA, radiofrequency ablation; TACE, transarterial chemoembolization; TAI, transarterial infusion chemothera.

Figure 5 summarizes the results of the standardized meta-analysis for 1-year OS. Overall, the TACE + 3DCRT vs TACE group was associated with a significant increase in 1-year OS (2.87, 2.24–3.69) with no heterogeneity (*P* = 0.000, *I*^2^ = 0.0%), and the TACE + sorafenib vs TACE group was associated with an increase tendency in 1-year OS (1.64, 0.96–2.79) with substantial heterogeneity (*P* = 0.034, *I*^2^ = 61.7%). A similar tendency could also be found in the TACE + PEI vs TACE group (2.20, 0.63–7.69; *P* = 0.156, *I*^2^ = 42.6%), DEB-TACE vs TACE group (1.06, 0.67–1.68; *P* = 0.440, *I*^2^ = 0.0%), TACE + PMCT vs TACE group (2.00, 0.90–4.44; *P* = 0.215, *I*^2^ = 35.1%), TACE + RFA vs RFA group (2.40, 0.99–5.80; *P* = 0.001, *I*^2^ = 73.4%), while the opposite tendency could be found in the TACE vs TAI group (0.95, 0.53–1.73; *P* = 0.431, *I*^2^ = 0.0%). The network and standardized meta-analysis results both revealed that TACE + 3DCRT has a significant, positive effect. The comparison-adjusted funnel plot of 1-year OS was not suggestive of any publication bias (Supplementary Figure 2). What’s more, some evidence of bias could be found in Begg’s (*P* = 0.020) and Egger’s (*P* = 0.000) tests, with moderate quality evidence according to the GRADE assessment (Supplementary Table 3).

#### 2-year OS

The network meta-analysis suggested that, compared with TACE treatment alone, TACE+PAI was ranked best for improving 2-year OS (21.74, 0.02–2.21e+3), followed by TACE+PMCT (3.65, 1.83–7.31), TACE + RFA (12.73, 0.02–8.16e+3), TACE + sorafenib (3.10, 1.77–5.45), TACE + 3DCRT (2.92, 2.28–3.73),TACE + PEI (2.97, 1.46–6.02), RFA (9.46, 0.01–6.05e+3), PAI (9.16, 0.01–9.27e+3), TACE + IFN (2.22, 1.27–3.88), TACE + SBRT (1.73, 0.74–4.04), TACE + amiodarone (1.65, 0.34–8.08), TACE + RT (1.62, 0.77–3.43), DEB-TACE (1.28, 0.80–2.03), SIRT (0.96, 0.18–5.25), TAI (1.10, 0.59–2.06), and TACE + brivanib (0.85, 0.47–1.55).

**Figure 6:**
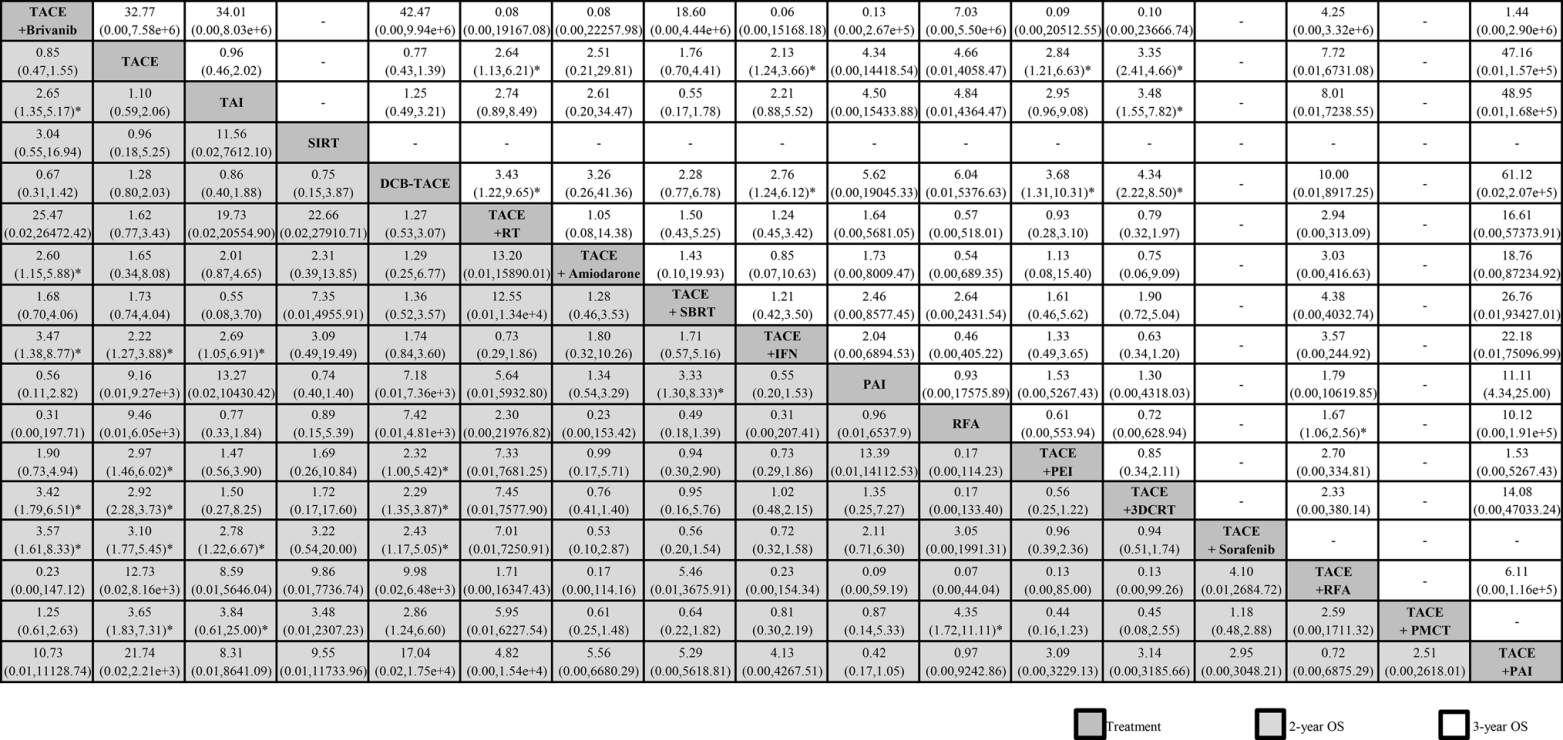
Network meta-analysis of 2-year OS and 3-year OS Treatments are reported in order of survival rate ranking according to SUCRA of treatment response. Comparisons should be read from left to right. The estimate is located at the intersection of the column-defining treatment and the row-defining treatment. For OS, an OR value above 1 favors the treatment arm. ^*^Result with significant difference. Abbreviations: 3DCRT, three—dimension eonformal radiotherapy; DEB-TACE, drug-eluting bead transarterial chemoembolization; IFN, interferon-ɑ; PAI, percutaneous acetic acid injection; PEI,percutaneous ethanol injection; PMCT, percutaneous microwave coagulation therapy; RFA, radiofrequency ablation; RT, radiotherapy therapy; SBRT, stereotactic body radiation therapy; SIRT, selective internal radiotherapy; TACE, transarterial chemoembolization; TAI, transarterial infusion chemotherapy.

When we assessed the comparative efficacy, TACE + PAI was superior to all other treatment arms. However, except for the TACE+PMCT arm, TACE + sorafenib arm, TACE + 3DCRT arm, TACE + PEI arm, and TACE + IFN arm, the other treatment arms did not reach significance. Treatment arms were comparable with each other for improving 2-year OS, with significant difference found in the TACE + sorafenib vs TACE + brivanib group (3.57, 1.61–8.33), TACE + 3DCRT vs TACE + brivanib group (3.42, 1.79–6.51), TACE + IFN vs TACE + brivanib group (3.47, 1.38–8.77), TACE + amiodarone vs TACE + brivanib group (2.60, 1.15–5.88), TAI vs TACE + brivanib group (2.65, 1.35–5.17), TACE+PMCT vs TAI (3.84, 0.61–25.00), TACE + sorafenib vs TAI (2.78, 1.22–6.67), TACE + IFN vs TAI (2.69, 1.05–6.91), TACE + sorafenib vs DEB-TACE (2.43, 1.17–5.05), TACE + 3DCRT vs DEB-TACE (2.29, 1.35–3.87), TACE + PEI vs DEB-TACE (2.32, 1.00–5.42), PAI vs TACE + SBRT (3.33, 1.30–8.33), and TACE + PMCT vs RFA (4.35, 1.72–11.11) (Figure 6).

#### 3-year OS

The network meta-analysis suggested that, compared with TACE treatment alone, TACE + PAI was ranked best for improving 3-year OS (47.16, 0.01–1.57e+5), followed by TACE + RFA (7.72, 0.01–6731.08), RFA (4.66, 0.01–4058.47), PAI (4.34, 0.00–1.44e+4), TACE + 3DCRT (3.35, 2.41–4.66), TACE + PEI (2.84, 1.21–6.63),TACE + RT (1.13, 2.28–6.21), TACE + amiodarone (2.51, 0.21–29.81), TACE + IFN (2.13, 1.24 to 3.66), TAI (0.96, 0.46–2.02), DEB-TACE (0.77, 0.43–1.39), and TACE + brivanib (32.77, 0.00–7.58e+6).

When we assessed the comparative efficacy, TACE + PAI was superior to all other treatment arms. However, except for the TACE + 3DCRT arm, TACE + PEI arm, TACE + IFN arm, and TACE + RT arm, the other treatment arms did not reach significance. Treatment arms were comparable with each other for improving 3-year OS, with significant differences found in the TACE + 3DCRT vs TAI group (3.48, 1.55–7.82), TACE + 3DCRT vs DEB-TACE group (4.34, 2.22–8.50), TACE + PEI vs DEB-TACE group (3.68, 1.31–10.31), TACE + RT vs DEB-TACE group (3.43, 1.22–9.65), and TACE + RFA vs RFA group (1.67, 1.06–2.56) (Figure 6).

### Secondary efficacy outcome - DFS

#### 1-year PFS

The network meta-analysis suggested that, compared with TACE treatment alone, TACE + PEI was ranked best for improving 1-year PFS (5.93, 1.39–25.34), followed by TACE + PAI (17.65, 0.00–2.23e+6), TACE + amiodarone (2.92, 0.53–16.16), TACE + RFA (3.05, 0.00–1.04e+5), PAI (5.92, 0.00–7.22e+6), SIRT (1.67, 0.21–13.52), RFA (1.72, 0.00–5.86e+4), TACE + sorafenib (1.31, 0.75–2.27), TACE + IFN (1.25, 0.52–2.96), DEB-TACE (1.18, 0.30–4.65), and TACE + brivanib (1.09, 0.51–2.33).

**Figure 7:**
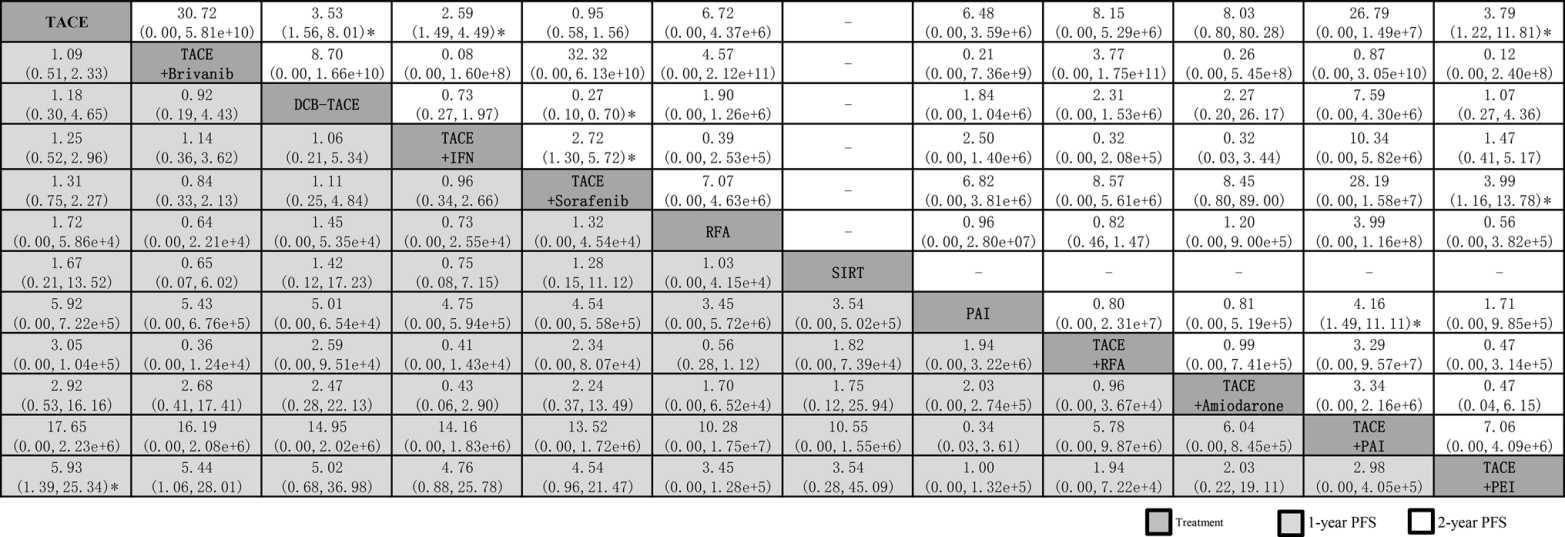
Network meta-analysis of 1-year PFS and 2-year PFS Treatments are reported in order of survival rate ranking according to SUCRA of treatment response. Comparisons should be read from left to right. The estimate is located at the intersection of the column-defining treatment and the row-defining treatment. For PFS, an OR value above 1 favors the treatment arm. ^*^Result with significant difference. Abbreviation: DEB-TACE, drug-eluting bead transarterial chemoembolization; IFN, interferon-ɑ; PAI, percutaneous acetic acid injection; PEI, percutaneous ethanol injection; RFA, radiofrequency ablation; SIRT, selective internal radiotherapy; TACE, transarterial chemoembolization.

When we assessed the comparative efficacy, TACE+PEI was superior to all other treatment arms. However, except for the TACE+PEI arm, the other treatment arms did not reach significance. Treatment arms were comparable to each other in terms of the effects of 1-year PFS, and no significant differences were found (Figure 7).

#### 2-year PFS

The network meta-analysis suggested that, compared with TACE treatment alone, TACE + brivanib was ranked best for improving 2-year PFS (30.72, 0.00–5.81e+10), followed by TACE + PAI (26.79, 0.00–1.49e+7), TACE + RFA (8.15, 0.00 to 5.29e+6), TACE + amiodarone (8.03, 0.80–80.28), RFA (6.72, 0.00–4.37e+6), PAI (6.48, 0.00–3.59e+6), TACE + PEI (3.79, 1.22–11.81), DEB-TACE (3.53, 1.56–8.01), TACE + IFN (2.59, 1.49–4.49), and TACE + sorafenib (0.95, 0.58–1.56).

When we assessed the comparative efficacy, TACE + brivanib was superior to all other treatment arms. However, except for the TACE + PEI arm, TACE + IFN arm, and DEB-TACE arm, the other treatment arms did not reach significance. Treatment arms were comparable with each other for improving 2-year PFS, with a significant difference found in the TACE + sorafenib vs TACE + IFN group (2.72, 1.30–5.72), TACE + PEI vs TACE + sorafenib group (3.99, 1.16–13.78), and TACE+PAI vs PAI group (4.16,1.49 to 11.11) (Figure 7).

### Safety outcomes

Table 3 summarizes the results of the toxicity associated with the different treatment groups. We found that combined therapy with sorafenib may elevate the AST level (5.541, 2.04–14.48) and ALT level (4.48, 1.66–12.11), and increase the occurrence of clinical symptoms, such as pain/abdominal distension, hand-foot-skin reaction, hypertension, and fatigue. Moreover, the TACE + RFA vs RFA group may increase the occurrence of nausea and vomiting (1.65, 1.01–2.69). The other treatment groups were safe, with mostly moderate- to high-quality evidence according to GRADE assessment.

**Table 3:** Grading of adverse events occurring in patients with unresectable hepatocellular carcinoma

Adverse Events	Treatment arms	All			Grade ≥ 3		
		Analysis (patients)	OR (95% CI)	*P*, *I*^2^	Analysis (patients)	OR (95% CI)	*P*, *I*^2^
***Hematological system***							
Leucopenia	TACE + 3DCRT vs TACE	3 (127/115)	0.76 (0.18, 3.16)	0.011, 77.6%	1 (40/36)	0.44 (0.04, 5.02)	-
Thrombocytopenia	TACE + 3DCRT vs TACE	1 (40/36)	0.56 (0.14, 2.15)	-	1 (40/36)	0.58 (0.09, 3.68)	-
	TACE + Sorafenib	2 (311/391)	4.12 (0.38, 44.69)	0.008, 85.7%	1 (229/227)	11.40 (1.46, 89.08)^*^	
***Clinical biochemistry***							
Total bilirubin	TACE +3DCRT vs TACE	1 (40/36)	3.43 (0.99, 11.85)	-			
AST	TACE + Sorafenib	1 (229/227)	5.541 (2.04, 14.48)^*^	-	1 (229/227)	4.13 (1.15, 14.83)^*^	-
ALT	TACE + 3DCRT vs TACE	2 (101/95)	1.80 (0.64, 5.03)	0.166, 48.0%	1 (40/36)	1.84 (0.16, 21.22)	-
	TACE + Sorafenib	1 (229/227)	4.48 (1.66, 12.11)^*^		1 (229/227)	4.07 (0.86, 19.39)	-
Albumin	TACE + 3DCRT vs TACE	1 (40/36)	3.43 (0.99, 11.85)	-	-		
***Clinical symptoms***							
Nausea/ vomiting	TACE + 3DCRT vs TACE	3 (127/115)	0.29 (0.05, 1.74)	0.001, 84.2%	1 (40/36)	0.88 (0.29, 2.64)	-
	TACE + Sorafenib	1 (81/81)	0.64 (0.28, 1.48)	-	-		
	TACE + RFA vs RFA	2 (141/142)	1.65 (1.01.2.69)^*^	0.902, 0.0%	2 (141/142)	3.03 (0.59, 15.55)	0.669, 0.0%
Fever	TACE + 3DCRT vs TACE	2 (114/108)	1.13 (0.62, 2.05)	0.910, 0.0%			
	TACE + RFA vs RFA	2 (141/142)	1.41 (0.85, 2.33)	0.911, 0.0%	2 (141/142)	2.41 (0.43, 13.69)	0.431, 0.0%
Pain/abdominal distension	TACE + 3DCRT vs TACE	2 (114/108)	1.38 (0.75, 2.55)	0.644, 0.0%			
	TACE + Sorafenib	2 (311/391)	29.00 (0.86, 983.24)	0.017, 82.5%	2 (311/391)	8.98 (1.61, 50.18)^*^	0.540, 0.0%
	TACE+RFA vs RFA	2 (141/142)	1.19 (0.53, 2.70)	-	2 (141/142)	1.00 (0.06, 16.47)	-
Upper gastrointestinal hemorrhage	TACE + Sorafenib	1 (82/164)	0.64 (0.24, 1.68)	-			
	TACE + RFA vs RFA	2 (141/142)	3.06 (0.31, 29.86)	1.000, 0.0%	-	-	-
Hand–foot–skin reaction.	TACE + Sorafenib	3 (392/472)	15.58 (0.77, 313.76)	0.000, 95.7%	2 (311/391)	31.40 (3.30, 299.23)^*^	0.275, 16.20%
Hypertension	TACE + Sorafenib	3 (392/472)	3.65 (1.10, 12.17)^*^	0.020, 74.3%	1 (229/227)	15.84 (2.08, 120.96)^*^	-
Fatigue	TACE + Sorafenib	1 (82.164)	107.92 (6.43, 1811.3)^*^				

^*^Results with significant differences. 3DCRT, three—dimension eonformal radiotherapy; RFA, radiofrequency ablation; TACE, transarterial chemoembolization.

## DISCUSSION

The network meta-analysis represents the most comprehensive synthesis of data for currently available data for the treatment for combination therapy with TACE with TACE monotherapy. We combined direct and indirect evidence from 48 clinical trials (50 analyses) comparing 19 different interventions on over five thousand patients with unresectable HCC to identify the best combination treatment with TACE. First, we found that most combination therapy arms were significantly more effective than TACE monotherapy in terms of treatment response. TACE + PEI had superior treatment efficacy, followed by TACE + sorafenib and TACE + 3DCRT (Figure 3). Second, TACE + 3DCRT was significantly more effective than TACE monotherapy in 6-month OS and 1-year OS (Figure 4), and similar results could be found in the standardized meta-analysis of 1-year OS (Figure 5). Third, we found that TACE + PAI and TACE + PMCT are the most significant effective combination therapies with TACE arms for 2-year OS, in comparison with other combination therapies and monotherapies; similar results could also be found for 3-year OS (Figure 6). Finally, TACE + PEI and TACE + PAI have displayed a leading efficacy tendency for 1-year PFS and 2-year DFS, in comparison with other treatment arms (Figure 7). Moreover, when considering the safety results, sorafenib and RFA may increase the occurrence of adverse effects (Table 3). In summary, in comparison with other types of combination therapy arms, TACE + 3DCRT, TACE + PEI, TACE + PMCT, TACE + PAI, and TACE + sorafenib were the more effective methods; however, because the adverse effects of sorafenib were serious, the use of TACE + sorafenib arm is not recommended.

This study extends the findings from primary clinical trials and previous meta-analyses by systematically synthesizing the efficacy data [28–75]. The meta-analysis differs from those in earlier studies in several ways. First, the main objective of the study was to identify the best combination treatment with TACE, including 19 arms, whereas the previous pairwise studies included only two combination treatments of TACE + RFA/PEI vs monotherapy [18, 25–26] with a standardized meta-analysis. Second, a network meta-analysis was used to directly and indirectly compare the treatment response, OS rate, and DFS rate, to determine the most appropriate types of combination therapy with TACE, while the previous article only considered the OS rate results [27]. Finally, the adverse effects of groups (*n* ≥ 2) were considered to observe the safety indicators.

This review followed the guidelines for conducting rigorous systematic reviews and network meta-analyses [56–58]. To identify as many relevant reports as possible, and to decrease the risk of bias, a comprehensive search strategy was designed. Based on these considerations, we observed no evidence of publication bias by statistical assessment. Combination therapy significantly increased the treatment response, 6-month OS, 1-year OS, 2-year OS, 3-year OS, 1-year DFS, and 2-year DFS (Figures 3–7). The results could be verified in the published review [14]. As for the safety section, sorafenib-associated adverse effects were more frequent in the combination therapy group, although it may improve the treatment response, OS, and DFS for unresectable HCC patients. The conclusions of the published articles [22, 79] are similar to those of our research. Moreover, DEB-TACE may have a higher OS rate and a higher DFS rate in patients with HCC than TACE. This conclusion can also be confirmed in published articles [80], which probably mean that DEB-TACE combined with other percutaneous local-ablation and targeted therapy drugs may further improve the survival rate.

This network meta-analysis had several limitations that merit further discussion. First, the review was restricted to trials involving patients with unresectable HCC. We excluded studies in which the patient was diagnosed with HCC, including a significant number of patients seen in the real world. Similarly, we excluded studies that only provided descriptive data. We did this to reduce heterogeneity and inconsistency among the trials included in this network meta-analysis; however, we acknowledge that this restricts the external validity of the results. Furthermore, in some cases, the original language of the publications could not be obtained, which reduced the number of trials we included and may have impacted the accuracy of the results. Furthermore, positive results are likely to be published, while negative results are not likely to be shared [81]. An additional limitation of standardize outcomes is their extensive heterogeneity (Figure 5), which indicated substantial variability in the outcomes of the included studies, although this was often because of the presence of heterogeneity in the baseline outcomes (Table 1) and differences observed in the trial design, Child-Pugh, and tumor stage of each study.

Additional clinical trials of combination therapy with TACE should include larger samples and be robust and randomized to confirm the effects and toxicity of combination therapy on patient-relevant or disease-specific outcomes, particularly in cancer patients with unresectable HCC. Future studies should ensure that appropriate methods are used for randomization, blinding, and intent to-treat. Furthermore, trials should assess outcomes using standardized or prescribed measures at similar time points. The analyses of individual data will be valuable for further exploration. More normative studies should be utilized in future network meta-analyses.

The findings of this comprehensive network meta-analysis provide some evidence that combination therapy with TACE may improve treatment response, 6-month OS, 1-year OS, 2-year OS, 3-year OS, 1-year DFS, and 2-year OS, which did not increase the occurrence of adverse effects, except sorafenib. On a local scale, patients with unresectable HCC should be encouraged to accept combination therapy with TACE, especially TACE + 3DCRT, TACE + PEI, TACE + PMCT, and TACE + PAI. In the clinical treatment of unresectable HCC patients, combination therapy with TACE can be used as the first-line treatment.

## MATERIALS AND METHODS

### Search strategy and selection criteria

This systematic review was performed with an a priori established protocol (PROSPERO CRD42017071102) [82], and the meta-analysis was performed following the Preferred Reporting Items for the Systematic Reviews and Meta-analyses (PRISMA) statement, the PRISMA network statement, and the Cochrane Collaboration recommendations [76–78].

We considered large-scale clinical trials of patients with unresectable HCC and searched PubMed, EMBase, the Cochrane Library, and Chinese National Knowledge Infrastructure for eligible trials form the very beginning of the databases to June 2017, comparing any of the following treatments: combination treatment with TACE, or TACE alone (see details in Supplementary Table 4). Trials of TACE or combined treatment groups within trials of TACE that did not have sufficient data for analysis were not considered. We also excluded trials published only as abstracts (with no additional data available from other sources). No language restrictions were applied. We then screened the reference lists of all obtained articles to avoid missing relevant trials.

We included the following treatment arms: three-dimensional conformal radiotherapy, drug-eluting bead transarterial chemoembolization, high-dose hepatic arterial infusion chemotherapy, interferon-ɑ, percutaneous acetic acid injection, percutaneous ethanol injection, percutaneous microwave coagulation therapy, radiofrequency ablation, radiotherapy therapy, stereotactic body radiation therapy, selective internal radiotherapy, transarterial chemoembolization, and transarterial infusion chemotherapy. Trials involving patients with a primary diagnosis of HCC, and no surgery can be made.

### Data extraction and quality assessment

Two investigators (XH and YHP) selected trials independently, and the third investigator (ZG or WHM) independently reviewed the main reports and supplementary materials and extracted the relevant information from the included trials with the predefined data extraction sheet. Data on efficacy and safety were abstracted from original studies. We extracted trial design, trial size, tumor stage, tumor size, Child-Pugh, ECOG status, HBV, HCV, the number of tumors, the details of treatment arms, the type of outcome (efficacy and safety), and outcome data for each time-point of interest. Whenever necessary, we approximated the means and measures of dispersion from figures in the original studies [83]. We extracted the results from intention-to-treat analyses whenever possible.

The risk of bias of the individual studies was assessed using the Newcastle-Ottawa scale score [84]. The scale is based on the Newcastle-Ottawa scale’s “yes” or “no” answers to the following criteria: (1) Is the case definition adequate? (2) Is there representativeness of the cases? (3) Is there selection of controls? (4) Is there a definition of controls? (5) Is there comparability of cases and controls? (6) Is there ascertainment of exposure? (7) Is the same method of ascertainment used for cases and controls? (8) Is there a non-response rate? A system analysis of studies was performed that excluded those with scores less than 5. Any discrepancies were resolved by consensus and arbitration by a panel of investigators within the review team.

### Outcomes

The primary efficacy outcome was treatment response. Local tumor response was measured according to the modified criteria for response evaluation in solid tumors (mRECIST) [85]; mRECIST defined the treatment response into four main categories: complete response (CR), partial response (PR), progressive disease (PD), and stable disease (SD). CR corresponds to disappearance of any intra-tumoral arterial enhancement in all target lesions, and PR corresponds to at least a 30% decrease in the sum of diameters of viable (enhancement in the arterial phase) target lesions, taking as a reference the baseline sum of the diameters of target lesions. PD is defined as an increase of at least 20% in the sum of the diameters of viable target lesions, taking as a reference the smallest sum of the diameters of viable target lesions recorded at the start of treatment; SD is defined as any cases that do not qualify for either PR or PD.

We also considered the 6-month, 1-year, 2-year, and 3-year overall survival (OS) as the primary efficacy outcomes of unresectable HCC associated with different treatment arms with or without TACE use. Our secondary efficacy outcome was 1-year to 2-year disease-free survival (DFS) associated with different treatment arms.

Our primary safety outcomes were hematological system and clinical biochemistry toxicity; the former including leucopenia and thrombocytopenia and the latter including total bilirubin, AST, and ALT, which could be stratified by all adverse events or grade ≥ 3. Our secondary safety outcomes were clinical symptoms toxicity, including nausea/vomiting, fever, pain/abdominal distension, upper gastrointestinal hemorrhage, hand–foot–skin reaction, hypertension, fatigue, and stomatitis, which also could be stratified by all adverse events or grade ≥ 3.

### Data synthesis and statistical analysis

We defined studies reporting multiple treatments and controls as sub-studies (marked as a/b) to avoid double-counting and mistreating data. First, a direct meta-analysis was performed with random-effects models because they are likely the most appropriate and conservative methodology to account for between-trial heterogeneity for each comparison [86–87]. To estimate pooled odds ratios (ORs) and 95% confidence intervals (95% CIs) heterogeneity was incorporated within and between studies, with STATA v14.0. Statistical heterogeneity was assessed with *P* values and *I*2 statistics, with values higher than 50% indicating substantial heterogeneity [88]. We also plotted a comparison-adjusted funnel plot for the network meta-analysis to detect the presence of any dominant publication bias in the network meta-analysis.

Second, we conducted a random-effects network meta-analysis using STATA v14.0. We summarized the results of the network meta-analysis with OR and their credible intervals (CrI) [89]. A common heterogeneity parameter was assumed for all comparisons; we also assessed global heterogeneity using *P* values and the *I*^2^ statistic.

The relative efficacy and safety of each treatment resulted from the combination of the direct evidence between the two treatment arms and the indirect evidence derived from the network meta-analysis, which are assumed to be coherent [86]. Inconsistency between direct and indirect sources of evidence was statistically assessed globally (by comparison of the fit and parsimony of consistency and inconsistency models) and locally (by calculation of the difference between direct and indirect estimates in all closed loops in the network) [90]. When a direct connection between two treatment arms was not available, the result was obtained only from indirect evidence.

We estimated the ranking probabilities for all treatments of being at each possible rank for each treatment arm. The treatment hierarchy was summarized and reported as the surface under the cumulative ranking curve (SUCRA) [91], ranging from 1, indicating that the treatment has a high likelihood of being best, to 0, indicating that the treatment has a high likelihood of being worst. A high SUCRA score corresponds to a higher ranking of survival rate from cancer compared with other treatments.

### Quality of evidence

We assessed the quality of evidence for our primary outcomes according to the Grading of Recommendations Assessment, Development and Evaluation (GRADE) system using GRADEpro GDT [92–93]. The GRADE system assesses risk of bias (study limitations), imprecision, inconsistency, indirectness of study results, and publication bias (classifying each as high, moderate, low, or very low) across the body of evidence to derive an overall summary of the quality of evidence.

### Patient involvement

No patients were involved in setting the research question or the outcome measures, nor were they involved in developing plans for the design or implementation of the study. No patients were asked for advice on the interpretation or presentation of results. There are no plans to disseminate the results of the research to study participants or the relevant patient community.

## SUPPLEMENTARY MATERIALS FIGURES






