# Supplementary material for: What is the best combination treatment with transarterial chemoembolization of unresectable hepatocellular carcinoma? a systematic review and network meta-analysis
Source: Oncotarget. 2017 Aug 10;8(59):100508–23. doi: 10.18632/oncotarget.20119 (PMC5725039; doi:10.18632/oncotarget.20119)
Supplement: Supplementary file 1 [file oncotarget-08-100508-s001.pdf]

What is the best combination treatment with transarterial chemoembolization of unresectable hepatocellular carcinoma? a systematic review and network meta-analysis

SUPPLEMENTARY MATERIALS

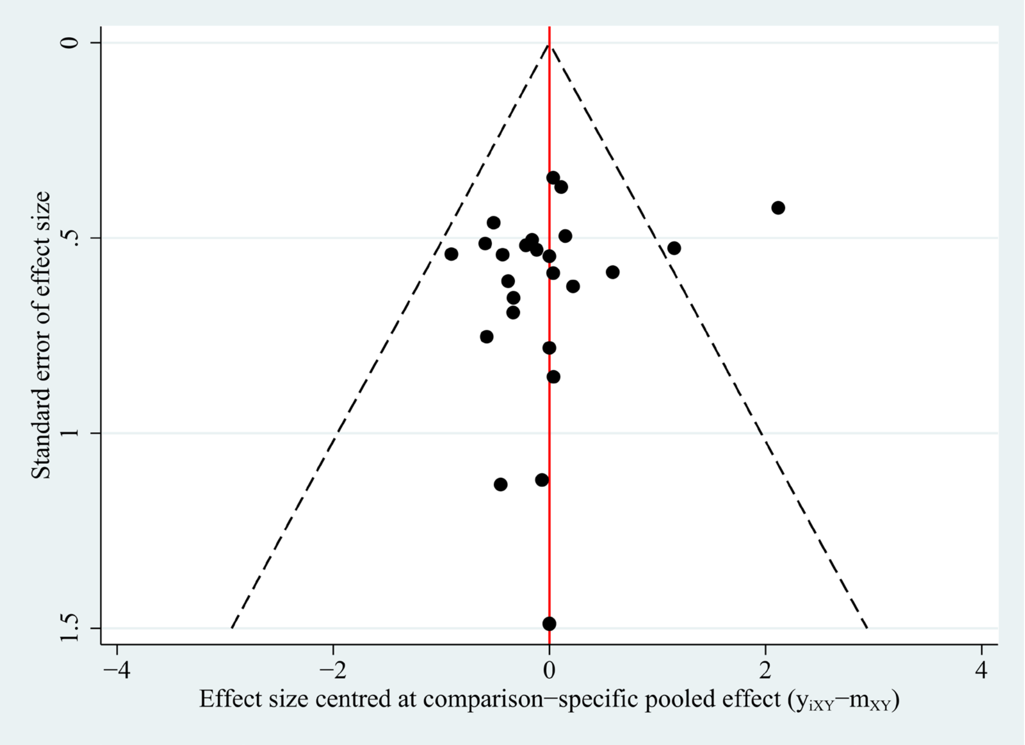

Supplementary Figure 1: Network funnel plots of music on treatment response.

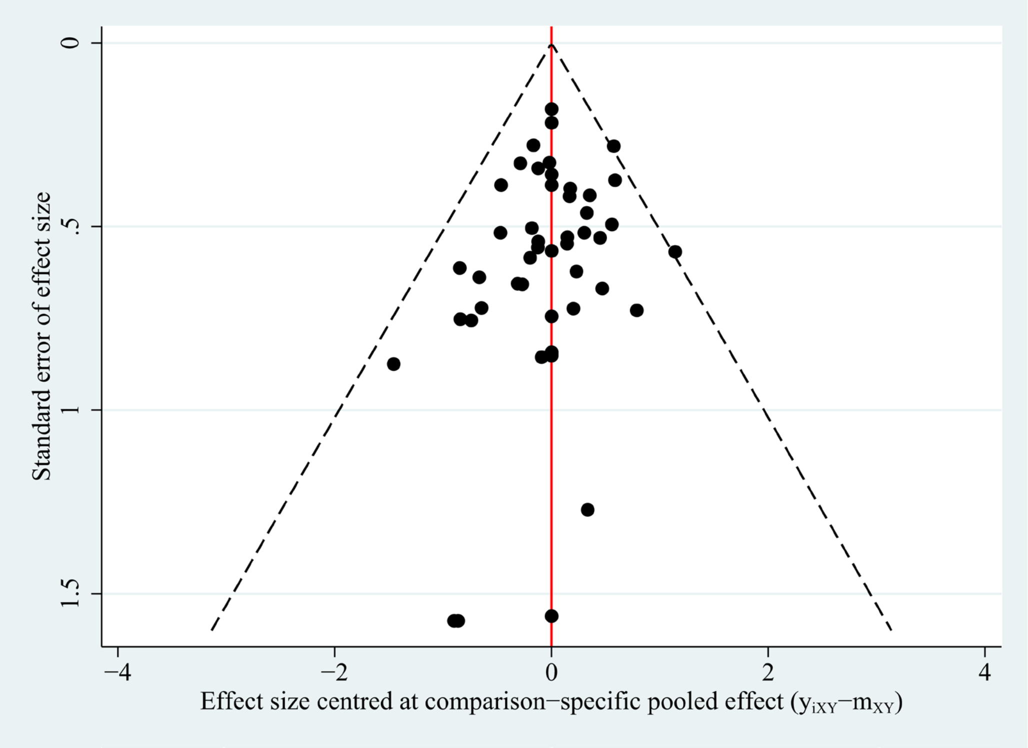

Supplementary Figure 2:Network funnel plots of music on 1-year overall survival rate.

Supplementary Table 1: Study Characteristics. See Supplementary_Table_1

Supplementary Table 2: Risk of Bias Assessments. See Supplementary_Table_2

Supplementary Table 3: Search strategies for pubmed, EMBASE and the cochrane library database

Search strategies for PubMed

#1.(Transarterial[All Fields] AND chemoembolization[All Fields] AND unresectable[All Fields] AND ("carcinoma, hepatocellular"[MeSH Terms] OR ("carcinoma"[All Fields] AND "hepatocellular"[All Fields]) OR "hepatocellular carcinoma"[All Fields] OR ("hepatocellular"[All Fields] AND "carcinoma"[All Fields]))) AND Clinical Trial[ptyp]

#2. (Transarterial[All Fields] AND chemoembolization[All Fields] AND unresectable[All Fields] AND ("carcinoma, hepatocellular"[MeSH Terms] OR ("carcinoma"[All Fields] AND "hepatocellular"[All Fields]) OR "hepatocellular carcinoma"[All Fields] OR ("hepatocellular"[All Fields] AND "carcinoma"[All Fields]))) AND Randomized Controlled Trial[ptyp]

#3. (Transarterial[All Fields] AND chemoembolization[All Fields] AND unresectable[All Fields] AND HCC[All Fields]) AND (Randomized Controlled Trial[ptyp] OR Clinical Trial[ptyp])

#4.(TACE[All Fields] AND unresectable[All Fields] AND ("carcinoma, hepatocellular"[MeSH Terms] OR ("carcinoma"[All Fields] AND "hepatocellular"[All Fields]) OR "hepatocellular carcinoma"[All Fields] OR ("hepatocellular"[All Fields] AND "carcinoma"[All Fields]))) AND (Randomized Controlled Trial[ptyp] OR Clinical Trial[ptyp])

Search strategies for EMbase

#1transarterial AND ('chemoembolization'/exp OR chemoembolization) AND unresectable AND hepatocellular AND ('carcinoma'/exp OR carcinoma)

#2 tace AND unresectable AND hepatocellular AND carcinoma

#3 transarterial AND chemoembolization AND unresectable AND hcc

Search strategies for Cochrane library

#1.unresectable hepatocellular carcinoma, Transarterial chemoembolization

#2. MeSH descriptor

Supplementary Table 4: Assessment of the quality of evidence using the grading of recommendations assessment

Number of analyses, by outcome	Quality assessment					Overall quality of evidence
	Risk of bias	Inconsistency	Indirectness	Imprecision	Other consideration	
**6-month OS**	Serious*	Serious†	Serious‡	Not serious	None	**+— Very Low*†‡**
**1-year OS**	Serious*	Serious†	Serious‡	Not serious	None	**+—Very Low*†‡**
**2-year OS**	Serious*	Serious†	Serious‡	Not serious	None	**+— Very Low*†‡**
**3-year OS**	Serious*	Serious†	Serious‡	Not serious	None	**+— Very Low*†‡**
**1-year DFS**	Serious*	Serious†	Serious‡	Not serious	None	**+— Very Low*†‡**
**2-year DFS**	Serious*	Serious†	Serious‡	Not serious	None	**+— Very Low*†‡**

*Not all studies are randomized controlled trials. †Substantial heterogeneity. ‡Network meta-analysis.
