# Supplementary material for: What is the best combination treatment with transarterial chemoembolization of unresectable hepatocellular carcinoma? a systematic review and network meta-analysis
Source: Oncotarget. 2017 Aug 10;8(59):100508–23. doi: 10.18632/oncotarget.20119 (PMC5725039; doi:10.18632/oncotarget.20119)
Supplement: Supplementary file 2 [file oncotarget-08-100508-s002.doc]

**Supplementary Table 1: Study Characteristics.**

| **Publication, Year, Location** | **Subjects** | | | | | | | | | **Design** | | |
| --- | --- | --- | --- | --- | --- | --- | --- | --- | --- | --- | --- | --- |
|  | **Design** | **Participants(N,age,male%)** | **Tumor stage(I-II/III-IV)** | **Tumor**  **size(cm)**  **(<5/5-/ <10/10-)** | **Child-Pugh**  **(A/B-C)** | **ECOG status (0/1)** | **HBV(+/-)** | **HCV(+/-)** | Number of tumors(single/[multiple](../../../../E:/Program%20Files%20(x86)/Youdao/Dict/7.1.0.0421/resultui/dict/javascript:%3B)) | **Arm I (combined with TACE)** | **Arm II** | **Follow-up** |
| Rahman FA, 2016, Malaysia [28] | CCT | I:45,63±13,80%  II:34,61±10,76.5% | - | - | - | - | I:14/41  II:13/21 | I:5/40  II:4/30 | I:10/35  II:17/17 | DCB-TACE | TACE | 2 years |
| Yao X , 2016,China[29] | CCT | I:50,56.5±12.2,88%  II:100,55.9±10.9,87% | - | - | I:42/8  II:86/14 | I:21/29  II:34/66 | I:42/8  II:83/17 | I:2/48  II:4/96 | - | TACE + Sorafenib | TACE | 2 years |
| Zhang XC, 2016, China[30] | CCT | I:44,-,70.45%  II:52,-,73.08% | - | - | I:43/1  II:48/4 | I:38/6  II:43/9 | I:41/3  II:47/5 | - | - | TACE + SBRT | TACE | 3 year |
| Kloeckner R, 2015, Germany [31] | CCT | I:76,-,89.9%  II:174,-,82.9% | - |  | I:51/25  II:103/71 | I:53/23  II:110/64 | I:10/66  II:14/160 | I:20/56  II:47/127 | I:73/3  II:169/5 | DCB-TACE | TACE | 5 years |
| Li M, 2015, China [32] | Single-center, CCT | I:70,  II:80,  53, 88% | - | - | 83/67 | - | 124/26 | - | - | TACE + PMCT | TACE | 2 years |
| Liu B, 2015, China [33] | RCT | I:70,-,86.7%  II:69,-,84.6% | - | I:40/30  II:38/31 | I:42/28  II:40/29 | - | I:60/10  II:58/11 |  | I:37/33  II:34/35 | TACE | TAI | 1 years |
| Ma J, 2015, China[34] | Multi-center, CCT | I:167; 52.19±11.83; 84.83%  II:174; 51.32±12.89; 98.85% | I:0/167  II:0/174 | - | I:146/26  II:172/2 | - | - | - | - | TACE+Licartin | TACE | 1 years |
| Pitton MB, 2015, Germany [35] | Single-center, RCT | I:12; 70.5±9.0; 83.33%  II:12; 71.8±7.2; 66.67% | I: 6/6  II:6/6 | - | I:10/2  II:9/3 | - | - | - | - | DCB-TACE | SIRT | 2 years |
| Yu Y,2015,China[36] | Single-center, CCT | I(a):20, 47.6±7.2; 65%  I(b):20, 48.1±8.0; 70%  II:20, 47.6±7.2; 65% | - | - | - | - | - | - | - | TACE+RFA | RFA | 2 years |
| Kudo M, 2014, Japan[37] | Multi-center, CCT | I:249; 57;82.73%  II:253, 59; 85.38% | - | I:189/60  II:195/58 | I:239/10  II:231/22 | I:201/48  II:203/50 | I:158/91  II:168/85 | I:49/200  II:42/211 | I:91/158  II:83/170 | TACE+Brivanib | TACE | 2 years |
| Liu HD, 2014, China[38] | Single-center, CCT | I:25,64,84%  II:27,61,77.78% | I:22/3  II:24/3 | - | - | - | - | - | - | TACE+3DCRT | TACE | 2 years |
| Sun H, 2014, China[39] | Single-center, RCT | I:81; 54.5±7.9; 83.95%  II:81; 53.9±8.2; 81.48% | - | - | I:70/11  II:72/9 | - | - | - | - | TACE + Sorafenib | TACE | 1 years |
| Yi Y, 2014, China[40] | Single-center, RCT | I:47; 56.8±5.6; 78.72%  II:47; 55.9±5.4; 72.34% | - | I:22/25  II:20/27 | I:45/2  II:44/3 | - | I:42/5  II:41/6 | I:4/43  II:5/42 | I:36/11  II:37/10 | TACE+RFA | RFA | 5 years |
| Bai W, 2013, China[41] | Single-center, RCT | I:82; 54±13; 89%  II:164;52±12; 89% | - | - | I:63/19  II:115/49 | I:30/38  II:48/101 | I:72/10  II:147/17 | I:4/78  II:7/157 | I:34/48  II:79/85 | TACE + Sorafenib | TACE | 2 years |
| Kang J, 2013,China[42] | Single-center, RCT | I:61; 53; 85.24%  II:59;51; 83.05% | - | - | I:55/11  II:51/8 | - | I:48/13  II:45/14 | - | I:50/11  II:51/8 | TACE+3DCRT | TACE | 3 years |
| Peng ZW, 2013 China[43] | Single-center, RCT | I:94; 53.3±11; 79.79%  II:95;55.3±13.3;74.74% | - | I:43/51  II:46/49 | I:90/4  II:90/5 | - | I:85/9  II:83/12 | I:6/88  II:6/89 | I:62/32  II:27/28 | TACE+RFA | RFA | 3 years |
| Chai Q, 2012, China[44] | Single-center,,CCT | I:34; 58.9±9.2; 58.82%  II:30;57.9±8.1;76.67% | - | - | - | - | - | - | - | TACE+3DCRT | TACE | 3 years |
| Gao ZY, 2012, China[45] | Single-center,RCT | I:53,  II:49,  53.87±5.97, 62.75% | - | - | - | - | - | - | - | TACE+3DCRT | TACE | 3 years |
| Ma WJ, 2012, China[46] | Single-center,RCT | I:32; 53.8;75%  II:36;55.2; 83.33% | - | I:23/13  II:25/7 | I:13/23  II:11/21 | - | - | - | - | TACE + PMCT | TACE | 2 years |
| Boulin M, 2011, Japan[47] | Single-center,RCT | I:13; -;85%  II:14;-; 86% | - | I:7/6  II:6/8 | I:12/1  II:13/1 |  | - | - | - | TACE + Amiodarone | TACE | 2 years |
| Kudo M , 2011, Japan[48] | Multi-center,RCT | I:229; 69;76.0%  II:229;70; 73.4% | - | - | - | I:201/28  II:202/27 | I:47/182  II:52/177 | I:139/90  II:148/81 | - | TACE + Sorafenib | TACE | 2 years |
| Leng N, 2011, China[49] | Single-center,,CCT | I:50,  II:39,  52±15.4, 80% | - | - | - | - | - | - | - | TACE+3DCRT | TACE | 3 years |
| Liu H, 2011, China[50] | Single-center,,CCT | I:30,  II:30,  -, 58.43% | - | - | - | - | 71.67% | - | - | TACE+3DCRT | TACE | 2 years |
| Pawlik TM,2011,USA[51] | Multi-center,RCT | P:56  C:56 | - | - | - | - | - | - | - | TACE + Sorafenib | TACE | - |
| Sacco R, 2011, Italy [52] | Single-center,,RCT | I:33; 71.3±7.2; 69.7%  II:34;68.7±8.1;64.71% | - | - | I:29/4  II:25/9 | - | I:4/29  II:4/30 | I:22/11  II:25/9 | - | DCB-TACE | TACE | 2 years |
| Jiang HY, 2010, China[53] | Single-center,,RCT | I:30; 56;80.0%  II:30;58; 76.67% | I:1/29  II:1/29 | - | I:25/5  II:24/6 | - | - | - | - | TACE + Sorafenib | TACE | 1 year |
| Kim HY, 2010, Korea[54] | Single-center,,RCT | I:31; 55±9.2  II:36;53±10.9 | I:0/31  II:0/36 | - | I:20/11  II:23/3 |  | I:26/5  II:30/6 | I:1/30  II:3/33 | - | TACE | HAIC | 1 year |
| Lin XQ, 2010, China[55] | Single-center,,CCT | I:40; -; 55%  II:40;-;50% | I:24/16  II:25/15 | I:35/5  II:34/6 | I:15/25  II:14/26 | - | - | - | - | TACE+3DCRT | TACE | 3 years |
| Morimoto M, 2010, Japan [56] | Single-center,,RCT | I:19;70; 79%  II:18;-;50% | - | - | I:18/1  II:16/2 | I:12/7  II:12/6 | I:0/19  II:0/18 | I:17/2  II:16/2 | - | TACE+RFA | RFA | 3 years |
| Pan WH, 2010,China[57] | Single-center,,RCT | I:30,  II:33,  52, 73.02% | I:0/30  II:0/33 | - | - | - | - | - | - | TACE+3DCRT | TACE | 3 years |
| Zhu ZY, 2010,China[58] | Single-center,,CCT | I:45; 48.5±11.4; 91.11%  II:45;50.6±12.3;88.89% | I:38/7  II:42/3 | - | I:36/9  II:39/6 | - | - | - | - | TACE+3DCRT | TACE | 3 years |
| Li M,2009,China[59] | Single-center,,RCT | I:108; -; 71.3%  II:108;-;68.5% | - | I:50/58  II:51/57 | I:98/10  II:99/9 | I:81/27  II:84/24 | I:77/31  II:86/22 | - | I:49/59  II:149/59 | TACE+IFN | TACE | 2 years |
| Okusaka T, 2009, Japan [60] | Multi-center,RCT | I:79;65.0; 77.2%  II:82;67.0;85.4% | I:20/59  II:41/41 | - | - | I:76/3  II:77/5 | I:11/68  II:7/75 | I:57/22  II:60/22 | - | TACE | TAI | 5 years |
| Shibata T, 2009, Japan [61] | Single-center,RCT | I:46; 67.2±8.9; 67.39%  II:43;69.8±8.0;76.74% | - | - | I:32/14  II:33/10 | - | I:12/34  II:9/34 | I:32/14  II:30/13 | - | TACE+RFA | RFA | 4 years |
| Wang FH, 2009,China[62] | Single-center,RCT | I:23;  II:23;  53, 84.78% | - | - | 40/6 | - | - | - | - | TACE+3DCRT | TACE | 2 years |
| Wu NN, 2008,China[63] | Single-center,CCT | I:77; 50; 87.01%  II:130;50;91.54% | I:28/49  II:38/92 | I:18/59  II:20/110 | - | - | - | - | - | TACE+3DCRT | TACE | 3 years |
| Yan G, 2008,China[64] | Single-center,CCT | I:23; 52; 78.26%  II:22;52;86.36% | - | I:11/12  II:9/13 | - | - | - | - | - | TACE+3DCRT | TACE | 3 years |
| Yang P, 2008,China[65] | Single-center,RCT | I:24; 59.1±11.4; 75%  II(a):11;57.6±11.8;72.73%  II(b):12;61.0±10.4;66.67% | I:7/17  II(a):5/19  II(b):5/18 | - | I:8/7  II(a):10/5  II(b):11/6 | - | - | - | I:5/19  II(a):7/4  II(b):8/4 | TACE+RFA | II(a):TACE  II(b):RFA | 1.5 years |
| Zhan WH, 2008,China[66] | Single-center,CCT | I:23; 52; 78.26%  II:22;53;86.36% | - | - | - | - | - | - | - | TACE+3DCRT | TACE | 3 years |
| Shang Y, 2007,China[67] | Single-center,CCT | I:40; 52; 60%  II:36;54;66.67% | I:28/12  II:22/14 | I:26/14  II:20/16 | - | - | I:32/8  II:30/6 | - | - | TACE+3DCRT | TACE | 3 years |
| Wang YB, 2007,China[68] | Single-center,CCT | I:31; 52; 87.10%  II:30;53;93.33% | - | I:14/17  II:12/18 | - | - | - | - | - | TACE+3DCRT | TACE | 3 years |
| Wang XL, 2006,China[69] | Single-center,RCT | I:30;  II:30;  52.7, 90% | - | - | 48/12 | - | - | - | - | TACE+3DCRT | TACE | 2 years |
| Becker G,2005,Germany[70] | Multi-center,RCT | I:27; 64; 74.1%  II:25;63.6;84% | I:26/1  II:25/0 | - | I:17/10  II:22/3 | - | I:7/20  II:7/18 | - | - | TACE+PEI | TACE | 3 years |
| Liu MZ,2005,China[71] | Single-center,CCT | I:54; 51; 75.9%  II:60;49.6;70% | - | I:39/15  II:44/16 | I:40/14  II:43/17 | - | - | - | - | TACE+RT | TACE | 3 years |
| Huo TI, 2003, China[72] | Single-center,CCT | I:53; 67±10;76.9%  II:55;68±9;75% | - | I:24/29  II:31/24 | I:38/15  II:29/26 |  | I:32/21  II:34/21 | - | I:21/32  II:30/25 | TACE+PAI | PAI | 3 years |
| Xu GH,2002, China[73] | Single-center,RCT | I:23  II:22 | - | - | - | - | - | - | - | TACE+PEI | TACE | 3 years |
| Koda M,2001,Japan [74] | Single-center,RCT | I:26; 66.2±8.0;53.85%  II:26;66.4±6.6;69.23% | - | - | I:19/5  II:14/8 | - | I:3/23  II:4/22 | I:21/5  II:21/5 | I:16/10  II:15/11 | TACE+PEI | TACE | 5 years |
| Bartolozzi C, 1995, Italy[75] | Single-center,RCT | I:26; 65.3±6.2;73.08%  II:27;66.1±4.9;81.48% | I:23/3  II:23/4 | - | I:19/5  II:21/8 | - | I:10/16  II:7/20 | I:19/7  II:21/6 | I:18/8  II:14/13 | TACE+PEI | TACE | 3 years |
