# Supplementary material for: What is the best combination treatment with transarterial chemoembolization of unresectable hepatocellular carcinoma? a systematic review and network meta-analysis
Source: Oncotarget. 2017 Aug 10;8(59):100508–23. doi: 10.18632/oncotarget.20119 (PMC5725039; doi:10.18632/oncotarget.20119)
Supplement: Supplementary file 3 [file oncotarget-08-100508-s003.doc]

**Supplementary Table 2: Risk of Bias Assessments**

Risk of Bias Assessment using the Newcastle-Ottawa Scale for Case-control Studies


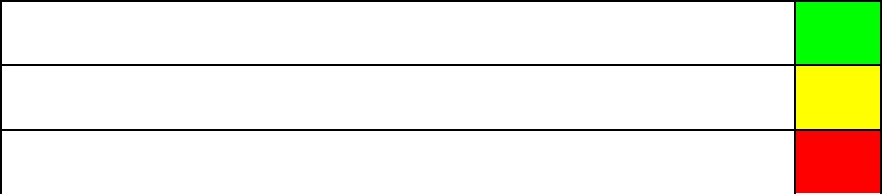


Low Risk of Bias

Intermediate or Unknown Risk of Bias

High Risk of Bias

| **Study** | **Is the case definition adequate** | **Representativeness of the cases** | **Selection of Controls** | **Definition of Controls** | **Comparability of cases and controls (/2)** | **Ascertainment of exposure(/2)** | **Same method of ascertainment for cases and controls** | **Non-Response rate** | **Overall rating and TOTAL SCORE / 10** |
| --- | --- | --- | --- | --- | --- | --- | --- | --- | --- |
|  |  |  |  |  |  |  |  |  |  |
| Rahman FA, 2016, Malaysia [28] | 1 | 1 | 1 | 1 | 2 | 0 | 1 | 1 | 8 |
| Yao X , 2016,China[29] | 1 | 1 | 1 | 1 | 2 | 0 | 1 | 1 | 8 |
| Zhang XC, 2016, China[30] | 1 | 1 | 1 | 1 | 2 | 0 | 1 | 1 | 8 |
| Kloeckner R, 2015, Germany [31] | 1 | 1 | 1 | 1 | 2 | 0 | 1 | 1 | 8 |
| Li M, 2015, China [32] | 1 | 1 | 1 | 1 | 2 | 0 | 1 | 1 | 8 |
| Liu B, 2015, China [33] | 1 | 1 | 1 | 1 | 2 | 1 | 1 | 1 | 9 |
| Ma J, 2015, China[34] | 1 | 1 | 1 | 1 | 2 | 0 | 1 | 1 | 8 |
| Pitton MB, 2015, Germany [35] | 1 | 1 | 1 | 1 | 2 | 0 | 1 | 1 | 8 |
| Yu Y,2015,China[36] | 1 | 1 | 1 | 1 | 2 | 0 | 1 | 1 | 8 |
| Kudo M, 2014, Japan[37] | 1 | 1 | 1 | 1 | 2 | 1 | 1 | 1 | 9 |
| Liu HD, 2014, China[38] | 1 | 1 | 1 | 1 | 2 | 0 | 1 | 1 | 8 |
| Sun H, 2014, China[39] | 1 | 1 | 1 | 1 | 2 | 0 | 1 | 1 | 8 |
| Yi Y, 2014, China[40] | 1 | 1 | 1 | 1 | 2 | 1 | 1 | 1 | 9 |
| Bai W, 2013, China[41] | 1 | 1 | 1 | 1 | 2 | 1 | 1 | 1 | 9 |
| Kang J, 2013,China[42] | 1 | 1 | 1 | 1 | 2 | 0 | 1 | 1 | 8 |
| Peng ZW, 2013 China[43] | 1 | 1 | 1 | 1 | 2 | 1 | 1 | 1 | 9 |
| Chai Q, 2012, China[44] | 1 | 1 | 1 | 1 | 2 | 0 | 1 | 1 | 8 |
| Gao ZY, 2012, China[45] | 1 | 1 | 1 | 1 | 2 | 0 | 1 | 1 | 8 |
| Ma WJ, 2012, China[46] | 1 | 1 | 1 | 1 | 2 | 0 | 1 | 1 | 8 |
| Boulin M, 2011, Japan[47] | 1 | 1 | 1 | 1 | 2 | 0 | 1 | 1 | 8 |
| Kudo M , 2011, Japan[48] | 1 | 1 | 1 | 1 | 2 | 1 | 1 | 1 | 9 |
| Leng N, 2011, China[49] | 1 | 1 | 1 | 1 | 2 | 0 | 1 | 1 | 8 |

| Liu H, 2011, China[50] | 1 | 1 | 1 | 1 | 2 | 0 | 1 | 1 | 8 |
| --- | --- | --- | --- | --- | --- | --- | --- | --- | --- |
| Pawlik TM,2011,USA[51] | 1 | 1 | 1 | 1 | 2 | 0 | 1 | 1 | 8 |
| Sacco R, 2011, Italy [52] | 1 | 1 | 1 | 1 | 2 | 0 | 1 | 1 | 8 |
| Jiang HY, 2010, China[53] | 1 | 1 | 1 | 1 | 2 | 0 | 1 | 1 | 8 |
| Kim HY, 2010, Korea[54] | 1 | 1 | 1 | 1 | 2 | 0 | 1 | 1 | 8 |
| Lin XQ, 2010, China[55] | 1 | 1 | 1 | 1 | 2 | 0 | 1 | 1 | 8 |
| Morimoto M, 2010, Japan [56] | 1 | 1 | 1 | 1 | 2 | 0 | 1 | 1 | 8 |
| Pan WH, 2010,China[57] | 1 | 1 | 1 | 1 | 2 | 0 | 1 | 1 | 8 |
| Zhu ZY, 2010,China[58] | 1 | 1 | 1 | 1 | 2 | 0 | 1 | 1 | 8 |
| Li M,2009,China[59] | 1 | 1 | 1 | 1 | 2 | 0 | 1 | 1 | 8 |
| Okusaka T, 2009, Japan [60] | 1 | 1 | 1 | 1 | 2 | 0 | 1 | 1 | 8 |
| Shibata T, 2009, Japan [61] | 1 | 1 | 1 | 1 | 2 | 0 | 1 | 1 | 8 |
| Wang FH, 2009,China[62] | 1 | 1 | 1 | 1 | 2 | 0 | 1 | 1 | 8 |
| Wu NN, 2008,China[63] | 1 | 1 | 1 | 1 | 2 | 0 | 1 | 1 | 8 |
| Yan G, 2008,China[64] | 1 | 1 | 1 | 1 | 2 | 0 | 1 | 1 | 8 |
| Yang P, 2008,China[65] | 1 | 1 | 1 | 1 | 2 | 0 | 1 | 1 | 8 |
| Zhan WH, 2008,China[66] | 1 | 1 | 1 | 1 | 2 | 0 | 1 | 1 | 8 |
| Shang Y, 2007,China[67] | 1 | 1 | 1 | 1 | 2 | 0 | 1 | 1 | 8 |
| Wang YB, 2007,China[68] | 1 | 1 | 1 | 1 | 2 | 0 | 1 | 1 | 8 |
| Wang XL, 2006,China[69] | 1 | 1 | 1 | 1 | 2 | 0 | 1 | 1 | 8 |
| Becker G,2005,Germany[70] | 1 | 1 | 1 | 1 | 2 | 0 | 1 | 1 | 8 |
| Liu MZ,2005,China[71] | 1 | 1 | 1 | 1 | 2 | 0 | 1 | 1 | 8 |
| Huo TI, 2003, China[72] | 1 | 1 | 1 | 1 | 2 | 0 | 1 | 1 | 8 |
| Xu GH,2002, China[73] | 1 | 1 | 1 | 1 | 2 | 0 | 1 | 1 | 8 |
| Koda M,2001,Japan [74] | 1 | 1 | 1 | 1 | 2 | 0 | 1 | 1 | 8 |
| Bartolozzi C, 1995, Italy[75] | 1 | 1 | 1 | 1 | 2 | 0 | 1 | 1 | 8 |
